# The Diagnosis of Facial Erysipelas

**Published:** 1908-12-26

**Authors:** 


### SPECIAL ARTICLE.

### THE DIAGNOSIS OF FACIAL ERYSIPELAS.

The diagnosis of erysipelas, particularly facial erysipelas, is not always easy; and mistakes are not at all infrequent even at the hands of the most experienced. The conditions which are most apt to be misdiagnosed as erysipelas are acute eczema; acute dermatitis, especially such as is produced by the use of lotions, hair-washes or hair-dyes; supra-orbital or infra-orbital herpes zoster; acute dacryocystitis; alveolar abscess, or other similar acute dental lesion; and sometimes even mumps.

It is usually taught that the most distinctive sign of erysipelas is its slightly raised, well-defined advancing margin. Such a well-defined raised edge to the reddened area may be seen with fair constancy in cases of erysipelas elsewhere, perhaps, but in the case of facial erysipelas the conditions seem to be quite different; at any rate, it is exceptional to find a well-marked raised border to the erysipelas area, and it therefore becomes necessary to consider other points in the differential diagnosis. Three such points have been very clearly described by M. G. Milian in the "Bulletins et Mémoires de la Société Médicale des Hôpitaux de Paris." He calls them: (1) Signe de la douleur à la pression; (2) Signe de l'oreille; (3) Signe du maximum centrifuge.

1. *Tenderness on pressure.*—That the erysipelatous area is tender upon pressure is mentioned in most books, but, as a rule, too little stress is laid upon it in practice. Erysipelas is never painless to pressure. If the finger-pulp is pressed even quite gently against the affected region, especially near its advancing edge, there is an almost reflex wincing and withdrawal of the head on the patient's part. He is unable, moreover, to lie with the affected part of his face or head in contact with the pillow.

In acute eczema, in supra-orbital or in infra-orbital herpes zoster, and in mumps, there may be subjective painfulness of the affected regions, but there is seldom any of the above acute tenderness upon digital palpation or pressure. In acute dacryocystitis, dental abscess and the like, there is pain which is both present all the time, and increased by digital pressure, but it is different from the pain and tenderness of erysipelas. Pressure causes pain in a limited area only, at the spot where the lesion is most intense. In the case of dacryocystitis this is at the internal angle of the orbit. In the case of acute inflammation secondary to a tooth the pain is most intense at a fixed spot, somewhere in the cheek at a level corresponding with the labio-gingival sulcus; the latter may often be seen partly filled up when the inside of the mouth is examined, the diagnosis being thereby cleared up.

The site of the pain and tenderness in erysipelas is, on the other hand, quite different; it is all over the inflamed area, with a maximum near the periphery when the erysipelas is at its height. This maximum point changes from one day to the next as the disease spreads in the way referred to below under the heading of "centrifugal maximum."

2. *The condition of the ear.*—Abscesses, subcutaneous inflammations of the cheek, and inflammatory swellings of the parotid glands always stop suddenly at the ear, leaving the auricle uninvolved. This is due to the close connection between the dermis and the perichondrium, especially over the outer aspect of the pinna. There is nowhere a great deal of cellular subcutaneous tissue upon the ear, and in some places there is none at all. It is on this account that nearly all subcutaneous inflammations, such as those secondary to teeth or the parotid glands, cease at the margins of the pinna. Erysipelas, on the other hand, is a dermatitis, spreading in the thickness of the skin itself without being dependent upon the subcutaneous tissues. It is able to spread to the pinna, therefore, with ease; and when it does so it usually spreads over the whole of it at once, causing it to swell up and become tense, red and shiny, and very tender and painful. This condition of the ear is highly characteristic; though taken by itself it might under some circumstances be mistaken for acute eczema, which may also involve the whole ear too. Eczema, however, is very likely to affect both ears at once, and in most cases the vesicular crust-forming character of the lesion will leave no doubt in the differential diagnosis.

3. *The centrifugal maximum.*—Erysipelas is a spreading complaint which clears up from the parts originally affected, whilst it invades adjacent healthy skin. It tends to advance ex-centrically. The result is that the maximum intensity of the lesion is always situated at a greater or less distance from the place where the trouble began, and it is nearly always near the periphery of the inflamed area of skin.

This sign is particularly useful in differentiating between erysipelas on the one hand, and such lesions as suppurative dacryocystitis, dental inflammations, or parotitis upon the other. These conditions are associated with a central maximum of swelling, redness, and pain. In dacryocystitis, for example, this swelling and redness have their maximum in the region of the lachrymal sac, decreasing the further one gets from the internal angle of the orbit. Next day and the day after the maximum inflammation remains still at the same spot. Quite the reverse is the case if erysipelas starts at the inner angle of the orbit; the maximum intensity of the mischief may at first be at the same spot as it would be for dacryocystitis, but within a short time it will have spread to the inferior eyelid and to the cheek, or else upwards on to the forehead. If there was doubt as to the differential diagnosis to-day, the doubt will have disappeared to-morrow in the face of the centrifugal change in the spot of maximum inflammation.

M. Milian very rightly emphasises one precaution that must be taken in gauging the centrifugal maximum, and that is in regard to the state of the eyelids themselves. The coverings are here so loose that œdema of them arises with the greatest ease. An œdema that would be barely visible in the cheek assumes enormous proportions when it reaches the eyelid, particularly the lower one. This must not be mistaken for the point of maximum inflammation. It is only the point of most obvious œdema. In other words, the degree of swelling of the eyelids should be almost neglected when one is forming a judgment as to where the point of maximum inflammation is, whether in a case of facial erysipelas or in one of the other diseases which may be mistaken for it.

